# Current Advances and Challenges in the Management of Cutaneous Squamous Cell Carcinoma in Immunosuppressed Patients

**DOI:** 10.3390/cancers16183118

**Published:** 2024-09-10

**Authors:** Sophie Li, Thomas Townes, Shorook Na’ara

**Affiliations:** The Department of Head and Neck Surgery, MD Anderson Cancer Center, University of Texas, Houston, TX 77030, USA; sli19@mdanderson.org (S.L.); ttownes@mdanderson.org (T.T.)

**Keywords:** cutaneous squamous cell carcinoma, immunosuppression, high-risk, immunotherapy, prophylaxis, Mohs micrographic surgery, chemotherapy, targeted therapy, radiation therapy

## Abstract

**Simple Summary:**

Cutaneous squamous cell carcinoma (cSCC) is a common and potentially dangerous skin cancer, especially for people with weakened immune systems, like those who have had organ transplants or certain blood cancers. These individuals are up to 100 times more likely to develop cSCC compared with the general population. This review discusses the current treatments for cSCC in these high-risk patients, emphasizing the importance of prevention and the variety of treatment options available. Using high-SPF sunscreen and certain medications can help reduce the chances of developing cSCC. Adjusting the medications that suppress the immune system can also lower the risk. Surgery remains the main treatment, with radiation therapy recommended for more serious cases. Other treatments such as chemotherapy and newer targeted therapies have been used, with mixed results. Immunotherapy, which helps the body’s own immune system fight the cancer, shows promise but needs more research to ensure it is safe for these vulnerable patients. This study highlights the need for future research to explore personalized treatment plans and combination therapies to improve outcomes for people with weakened immune systems.

**Abstract:**

Cutaneous squamous cell carcinoma (cSCC) is the second most common skin malignancy and poses a significant risk to immunosuppressed patients, such as solid organ transplant recipients and those with hematopoietic malignancies, who are up to 100 times more likely to develop cSCC compared with the general population. This review summarizes the current state of treatment for cSCC in immunosuppressed patients, focusing on prevention, prophylaxis, surgical and non-surgical treatments, and emerging therapies. Preventative measures, including high-SPF sunscreen and prophylactic retinoids, are crucial for reducing cSCC incidence in these patients. Adjusting immunosuppressive regimens, particularly favoring mTOR inhibitors over calcineurin inhibitors, has been shown to lower cSCC risk. Surgical excision and Mohs micrographic surgery remain the primary treatments, with adjuvant radiation therapy recommended for high-risk cases. Traditional chemotherapy and targeted therapies like EGFR inhibitors have been utilized, though their efficacy varies. Immunotherapy, particularly with agents like cemiplimab and pembrolizumab, has shown promise, but its use in immunosuppressed patients requires further investigation due to potential risks of organ rejection and exacerbation of underlying conditions. Treatment of cSCC in immunosuppressed patients is multifaceted, involving preventive strategies, tailored surgical approaches, and cautious use of systemic therapies. While immunotherapy has emerged as a promising option, its application in immunosuppressed populations necessitates further research to optimize safety and efficacy. Future studies should focus on the integration of personalized medicine and combination therapies to improve outcomes for this vulnerable patient group.

## 1. Introduction

Cutaneous squamous cell carcinoma (cSCC) is the second most common skin malignancy in the world after basal cell carcinoma, making up approximately 20% of all cases of skin cancer [1]. It is defined by invasive, abnormal proliferation of keratinocytes in the epidermis. The main environmental risk factor for cSCC is ultraviolet (UV) radiation from cumulative sun exposure as well as indoor tanning devices [2]. Immunosuppression is another major risk factor for cSCC. Solid organ transplant recipients have up to 200 times higher risk of developing cSCC compared with the general population [3]. Patients with chronic lymphoblastic leukemia have high incidence of cSCC as well, with a 3.66-fold increase in risk, and patients with HIV have a 2.76-fold increase [4,5]. Other immunocompromised patients including stem cell transplant recipients and those with other hematopoietic malignancies or chronic autoimmune disease also have increased risk.

In immunocompetent patients, the incidence of basal cell carcinoma (BCC) is greater than cSCC by a ratio of 4:1. The incidence of these lesions in the immunocompromised, however, is reversed, with a general 4:1 ratio of cSCC to BCC, though this may vary according to patient ethnicity and geographical location, or by etiology of immunosuppression [6]. For instance, studies on Mediterranean and South American populations show relatively higher predominance of BCC, and patients with HIV have an incidence ratio of BCC–cSCC in line with the general population [5,7,8].

Treating immunocompromised patients with cSCC involves significant clinical challenges. Immunosuppression is associated with more aggressive disease and worse survival outcomes. Studies have revealed that cSCC in immunosuppressed patients is more likely to present with poorly differentiated disease, multifocality, recurrence, and metastasis [4,6,9,10]. Patients also have significantly worse disease-specific survival compared with immunocompetent patients, with over twice the risk of disease-specific death [6]. By definition, these patients often have increased comorbidities compared with the immunocompetent population and, due to the propensity of their disease to be more aggressive, may require more intense treatment to achieve a cure [4]. This can bring challenges to their care involving tradeoffs between disease clearance and functional outcomes. Therefore, treatment of cSCC in immunosuppressed patients is often nuanced and multifaceted. In this review, we summarize the current state of treatment for cSCC in immunosuppressed patients. We begin by discussing genetic and molecular factors that provide insights into the increased risk of cSCC in the immunocompromised state, prevention and prophylactic strategies for these patients, and adjustments in immunosuppressive regimens that decrease risk of developing cSCC. We then discuss treatment methods and strategies for these lesions, first addressing treatment of premalignant lesions, followed by a summary of the modalities currently used to treat cSCC in the immunosuppressed patient, including surgery, radiation, chemotherapy, targeted therapy, and immunotherapy. 

## 2. Genetic and Molecular Insights

The genetic landscape of cSCC is characterized by a high mutational burden that primarily consists of deleterious mutations in tumor suppressor genes, caused by UV radiation [11,12]. A meta-analysis of the largest cohort of cSCC to date discovered possible driver mutations in 30 genes, including *NOTCH1/2*, *TP53*, *CDKN2A*, *ARID2*, *FAT1*, and *HRAS* [11]. In immunosuppressed patients, more specifically, the tumor mutational burden and genetic variants have been shown to be similar to those of immunocompetent patients [13,14]. However, in a study that analyzed 20 cSCC samples, 50% of the tumors from immunosuppressed patients exhibited microsatellite instability compared with only 17% of the samples from the immunocompetent patients [14]. Whole exome sequencing analysis has also revealed that azathioprine treatment is significantly associated with a unique mutation signature that may be associated with UVA phototoxicity [12]. Further studies are needed to investigate novel therapeutic agents that target these genetic aberrations.

The molecular pathways involved in cSCC are less well known. One of the key molecular targets in cSCC is epidermal growth factor receptor (EGFR), which is expressed in approximately 90% of cSCC [15]. EGFR is involved in signaling pathways that cause cell proliferation, apoptosis, invasion, angiogenesis, and metastasis [16]. The MEK/ERK signaling pathway has also been shown to be a promising target in mouse models, but further studies are needed to assess the safety and efficacy of this therapy for prevention and treatment of cSCC in humans [17]. Studies in immunosuppressed patients have revealed the potential involvement of the mammalian target of rapamycin (mTOR) pathway in cSCC development. Particularly in renal transplant recipients, mTOR inhibitors have demonstrated significantly reduced incidence of skin cancers compared with other immunosuppressants such as calcineurin inhibitors and cyclosporine [18,19].

The tumor microenvironment of cSCC in immunocompromised patients involves various alterations in molecular and genetic pathways that support the growth and proliferation of the aberrant cells. In solid organ transplant recipients, lymphocytes play a significant role in establishing an immunosuppressive environment in and around the lesions. Compared with immunocompetent patients, organ transplant recipients with cSCC have a lower tumor inflammatory infiltrate density and an increased level of circulatory Tregs [20]. Studies have shown that these patients have lower expression of CD4+ mRNA, reduced CD8+ T-cell infiltration, and an increased ratio of Treg to CD8+ T cells that is associated with decreased immunosurveillance [21,22,23]. Bottomley et al. found that an increase in the proportion of senescent CD8+ T cells was predictive of cSCC development and recurrence in renal transplant patients, as indicated by increased expression of the immune senescence marker CD57 [24]. Further exploration of these alterations in T cells may guide the development of novel immunotherapies for immunosuppressed patients. The tumor microenvironment in immunosuppressed patients is also significantly impacted by human papillomavirus (HPV). Organ transplant recipients have a significantly higher β-HPV load and activity compared with immunocompetent patients, which has been associated with an increased risk of cSCC [25,26]. Strickley et al. demonstrated that this is due to the lack of T-cell immunity in these patients rather than the oncogenic effect of the virus [26]. These findings suggest that boosting T-cell immunity against HPV may be a promising avenue for preventing the development of cSCC in immunosuppressed patients. 

## 3. Adjusting Immunosuppressive Regimens

In transplant recipients, immunosuppressive regimens intended to prevent graft failure and rejection play a major role in the development of cSCC. Therefore, adjusting immunosuppressive regimens to minimize their carcinogenic potential is generally advised for these patients. mTOR inhibitors such as sirolimus and everolimus have been associated with a lower incidence of cSCC and subsequent nonmelanoma skin cancers, compared with calcineurin inhibitors and other older immunosuppressants, in multiple studies [27,28,29,30,31]. In a case study of a heart transplant recipient with metastatic cSCC, the patient’s immunosuppressive regimen consisting of calcineurin inhibitor and mycophenolic acid was replaced with everolimus, which enhanced T-cell function and prevented graft rejection [32]. Mycophenolate mofetil (MMF) is another option for an immunosuppressant. Studies have demonstrated that MMF can reduce skin photosensitivity to UVA and reduce the risk of developing nonmelanoma skin cancers [29,33]. In contrast, azathioprine has a high carcinogenic potential as it causes skin hypersensitivity to UVA [33]. A retrospective study evaluating the association between immunosuppression regimens in organ transplant recipients and the risk of cSCC in heart and kidney transplant recipients found that patients who used azathioprine were twice as likely to develop cSCC [27].

## 4. Treatment of Premalignant Lesions

Given that immunosuppressed patients with cSCC are likely to present with more aggressive disease and have worse survival outcomes compared with immunocompetent patients, treatment of premalignant lesions to prevent the progression to cancer is imperative. Biopsy is often the first step to treating patients with premalignant lesions. A retrospective study demonstrated that 70% of cutaneous squamous cell carcinoma in situ (cSCC-IS) cases in immunocompetent patients cleared after biopsy but only 47% of cases were cleared in immunocompromised patients. This suggests that treatment of premalignant lesions in immunosuppressed patients requires additional measures.

Other modalities for treating cSCC-IS in immunosuppressed patients include topical 5-fluorouracil, imiquimod, photodynamic therapy, cryotherapy, curettage, laser, and standard surgical excision [34]. One study including both immunocompetent and immunosuppressed patients found that topical 5-fluorouracil as a primary treatment or after Mohs micrographic surgery as an adjuvant therapy both had over 90% clearance rates [35].

For actinic keratosis, treatment options include topical imiquimod, topical 5-fluorouracil, and cryosurgery [36]. Out of the various topical treatments available, 5-fluorouracil has been shown in immunocompetent patients to be the most effective at reducing the number of actinic keratosis lesions [37]. In a phase II randomized clinical trial, topical 5-fluorouracil was found to be more effective than imiquimod at clearance and prevention of actinic keratosis in solid organ transplant recipients [38]. Combination therapy is also emerging as a treatment option for patients. In comparison to monotherapy, photodynamic therapy combined with either topical imiquimod, 5-fluorouracil, ingenol mebutate, tazarotene, or calcipotriol resulted in higher clearance rates [39]. Cryotherapy was also more effective at clearing actinic keratosis when used in conjunction with topical treatments [40]. However, more research is necessary to determine whether these treatment options are effective and safe in immunosuppressed patients. 

## 5. Surgery

Surgery is the primary treatment for patients with cSCC. In the guidelines set forth by the American Academy of Dermatology, surgical excision is recommended for low-risk cSCC and Mohs micrographic surgery (MMS) is recommended for high-risk cases [1]. Since they tend to have more aggressive disease characteristics, MMS may be preferable for immunosuppressed patients as it allows intraoperative margin assessment [10]. This technique also allows better tissue conservation, which is a key consideration because 49% to 60% of cSCC cases occur in the head and neck where cosmetic and functional integrity can be significantly impacted [41]. MMS provides excellent local control rates, with recurrence in high-risk cSCC as low as 1.5% [42]. However, there are limitations to MMS. Particularly in patients with chronic lymphocytic leukemia, the presence of dense leukemic infiltrates can complicate the interpretation of histological sections and evaluation of tumor margins [43]. Immunosuppressed patients are also more likely to have postoperative complications such as infection and wound dehiscence compared with immunocompetent patients [44]. However, other studies found that immunosuppression was not a significant risk factor for surgical site infections and postoperative antibiotics did not significantly reduce the incidence of infection in these patients [45,46]. In cases where MMS is not an option, surgical excision may be appropriate [31]. Because cSCC in immunosuppressed patients tends to present with more aggressive histopathological characteristics, wider excision margins are necessary to ensure clearance of the primary tumor and prevent recurrence [31,47].

For patients with lymph node involvement, the National Cancer Comprehensive Network (NCCN) recommends neck dissection and postoperative radiation therapy depending on the number and size of the positive lymph nodes. Sentinel lymph node biopsy may be considered for patients with multiple risk factors, but it is unclear whether this leads to improved patient outcomes [31].

## 6. Radiation

Radiation therapy is typically not given to patients as a first-line treatment. Patients with cSCC in the head and neck have poorer outcomes when radiation therapy is used as the primary therapy, with locoregional recurrence rates of up to 30% in the presence of negative prognostic factors such as immunosuppression [42,48]. However, it may be an option in patients who are not surgical candidates or choose not to undergo surgery [1]. In addition, adjuvant radiation therapy is an important component of treatment for high-risk cSCC. The National Cancer Comprehensive Network recommends adjuvant radiation therapy for patients with positive margins after surgery and patients with aggressive clinicopathologic features indicative of poor prognosis such as large tumor diameter, perineural invasion, and multifocal nerve invasion [31]. While there is a lack of prospective randomized control trials investigating the efficacy of adjuvant radiation therapy, retrospective analyses suggest that it is beneficial for some higher-risk cases. Harris et al. demonstrated that adjuvant radiation therapy was associated with improved disease-free survival in advanced cSCC patients with perineural invasion and regional disease [49]. Studies have also found that adjuvant radiation therapy lowers recurrence risk in patients with a high T stage or parotid metastases [50,51]. However, a multi-institutional retrospective study revealed that despite the addition of postoperative radiation therapy, immunosuppressed patients experienced significantly lower 2-year locoregional recurrence-free survival and progression-free survival compared with immunocompetent patients, suggesting that immunosuppressed patients may require additional treatment in order to control their disease [10].

## 7. Traditional Chemotherapy and Targeted Therapy

Systemic therapy including traditional chemotherapy, targeted therapy, and immunotherapy is generally reserved for patients who are not candidates for surgical intervention and unresectable disease that is high-risk, recurrent, or metastatic (Table 1). A prospective phase II study with 21 patients who had unresectable cSCC in the head and neck showed that 63% of patients had a complete response to chemoradiotherapy with either cisplatin or carboplatin [52]. Given the increased risk of recurrence and multifocality with immunosuppressed patients, systemic therapy may be a treatment option for this population of patients, but data on their efficacy in immunocompromised patients are limited [10,53]. The studies that have been conducted with immunocompetent populations suggest that the addition of chemotherapy may be beneficial in high-risk cSCC. A systemic review revealed that in patients with metastatic cutaneous squamous cell carcinoma, there was an overall response rate of 45% with cisplatin treatment [54]. Tanvetyanon et al. found that patients with high-risk cSCC who were treated with adjuvant chemoradiation had significantly better recurrence-free survival compared with those who received adjuvant radiation therapy only [55]. However, there have also been a number of studies that have found no added benefit from adjuvant chemotherapy [56,57,58]. When administering chemotherapy agents to immunosuppressed patients, additional considerations must be made depending on the cause of their immunosuppression and the specific drug. Platinum-based compounds such as cisplatin and carboplatin can result in myelosuppression and nephrotoxicity [59]. Paclitaxel, a chemotherapy agent recommended for use in combination with other drugs, is also myelosuppressive and requires dose adjustments in cases of liver dysfunction [31,59].

Targeted therapy, particularly EGFR inhibitors, is another treatment option that has been investigated in recent years. Cetuximab, a monoclonal antibody against EGFR, was found to inhibit or arrest cell cycle growth of tumor cell lines in vitro and has been tested for use as targeted therapy in cSCC, with favorable side-effect profiles [60,61]. A phase II clinical trial demonstrated that first-line treatment with cetuximab in patients with unresectable cSCC was effective for disease control, with a 69% disease-control rate at 6 weeks from the start of treatment [62]. Another phase II trial investigating cetuximab as an adjuvant therapy to postoperative radiation therapy for locally advanced cSCC found that this treatment regimen achieved 91% locoregional control and 71% disease-free survival [63]. A systemic review consisting of nine cases of metastatic cSCC treated with cetuximab revealed that 78% of patients had an overall response and 67% had a complete response, which were both higher than with cisplatin treatment [54]. However, other studies have shown no improvement in outcomes with cetuximab compared with traditional chemotherapy [64,65]. Although there have not been any studies to date that have investigated the efficacy of EGFR inhibitors specifically in immunosuppressed patients, a recent retrospective study examined the response to cetuximab in patients with cSCC who were not candidates for immunotherapy or were refractory to immunotherapy [66]. In the cohort of patients who had never undergone immunotherapy, 80% of them were considered immunosuppressed. The overall response rate and disease-control rate for this group were both 80%, suggesting that cetuximab may be an effective alternative to immunotherapy for immunosuppressed patients [66]. Other EGRF inhibitors that have been studied as potential treatments for cSCC include gefitinib and panitumumab. In patients with incurable cSCC, gefitinib was shown to have only a 19% overall response rate [67]. Panitumumab has had better efficacy, especially when used in adjunct with radiation therapy, in prospective and retrospective studies [68,69].

**Table 1:** Summary of the studies investigating non-surgical treatment of cutaneous squamous cell carcinoma in immunosuppressed patients.

Author	Study Design	Cohort	Treatment	Results
Hanna et al. [70]	Nonrandomized trial	12 renal transplant recipients	Cemiplimab	46% response rate to the treatment with no kidney rejection or loss
Joo et al. [32]	Case study	1 heart transplant recipient	mTOR inhibitor prophylaxis + talimogene laherparepvec (T-VEC) injection	No allograft rejection occurred after treatment
Ali et al. [71]	Case study	1 renal transplant recipient	Cemiplimab	Complete disease remission with no allograft rejection after treatment
Schenk et al. [72]	Prospective trial	12 renal transplant recipient	Nivolumab + tacrolimus + prednisone ± ipilimumab	Tacrolimus and prednisone failed to provide sufficient allograft protection
Alloghbi et al. [73]	Case study	1 HIV patient	Cemiplimab	Complete response with no toxicities
Brereton et al. [74]	Case study	1 AIDS patient	Cemimplimab-rwlc	No signs or symptoms of metastatic disease

## 8. Immunotherapy

Immunotherapy has emerged in recent years as a prospective treatment option for advanced and unresectable cSCC. Compared with other systemic treatments including traditional chemotherapy and targeted therapy, immunotherapy has improved progression-free survival and overall survival in advanced cSCC [75]. The two immunotherapy agents that are currently recommended by the National Comprehensive Cancer Network are cemiplimab and pembrolizumab [31]. Cemiplimab, a human monoclonal antibody for the programmed death 1 (PD-1) protein, has been approved by the FDA for the treatment of patients with metastatic or locally advanced cSCC who are not candidates for curative surgery or radiation. Phase I and phase II clinical trials in immunocompetent patients that led to the FDA approval of the drug demonstrated significant efficacy, with notable response rates of 50% and 47%, respectively [76]. Cemiplimab, like other checkpoint inhibitors, has shown promise due to its ability to enhance the body’s immune response against tumor cells. Pembrolizumab is another FDA-approved anti-PD-1 monoclonal antibody. The approval was based on the KEYNOTE-629 trial, which showed an objective response rate (ORR) of 34% with pembrolizumab in these patients [77,78]. Immunocompetent patients with locally advanced cSCC had an overall response rate of 50% and patients with recurrent or metastatic cSCC had an overall response rate of 35%. The trial also highlighted that pembrolizumab could be administered at 200 mg every 3 weeks or 400 mg every 6 weeks, with safety profiles consistent with other uses of pembrolizumab. Nivolumab has also been shown to be effective in treating locally advanced or metastatic cSCC, but more studies with larger sample sizes are needed to characterize the utility of this drug [79]. 

Despite the promising evidence for immunotherapy in immunocompetent patients, the safety and efficacy of immunotherapy in immunosuppressed patients with cSCCs has not been studied extensively. A recent phase I clinical trial assessing cemiplimab in kidney transplant recipients with advanced cSCC found that it produced an observable response in 46% of patients, and none of the 12 patients in the cohort experienced organ rejection [70]. Case reports of successful treatment of advanced cSCC with immunotherapy in solid organ recipients have also been published in recent years [32,71]. Preliminary data from the C.A.S.E. study evaluating the clinical effectiveness of cemiplimab for advanced cSCC found that out of the 19 immunocompromised patients in the cohort, 47% had either a complete or partial response and 1 patient experienced organ transplant rejection [80]. However, other immunotherapy regimens have yielded less favorable results. A phase I/II clinical trial testing a combination of nivolumab, tacrolimus, and prednisone with or without ipilimumab in kidney transplant recipients with advanced skin cancers found that nivolumab failed to induce tumor regression, and three out of the eight patients experienced allograft loss due to the treatment [72]. A multicenter retrospective study found that among kidney transplant patients in the cSCC subgroup who were treated with immune checkpoint inhibitors, there was a 33.3% response rate and a 37.5% acute rejection rate [81]. In patients with accompanying hematological malignancies, nivolumab has also been shown to be effective in treating locally advanced and metastatic cSCC, with no significant difference in the occurrence of treatment-related adverse events compared with the immunocompetent patients that were included in the study [82]. However, a retrospective study found that the efficacy of immunotherapy was significantly lower in patients with concomitant hematological malignancies compared with immunocompetent patients [83]. Treatment of cSCC in patients living with HIV (PLWH) using immunotherapy has been limited to case studies, though those have shown promising outcomes [73,74]. A multi-institutional phase I study demonstrated that nivolumab had a similar safety profile and response rate in PLWH compared with immunocompetent patients, without any significant changes to viral HIV load or CD4 counts when it was used to treat Kaposi sarcoma and various solid tumors [84]. Additional studies in other types of cancer and immunotherapy agents further support the safety and therapeutic activity of those drugs in PLWH [85,86]. These results may extrapolate to cSCC, but additional testing is needed. Another subpopulation of immunosuppressed patients includes those with autoimmune conditions. There is limited evidence regarding the use of immunotherapy to treat cSCC in patients with autoimmune conditions. Based on studies conducted in other types of cancer, immunotherapy can cause exacerbation of preexisting autoimmune conditions and immune-related adverse events, but these can be managed with conventional therapies, including corticosteroids [87,88].

In addition to using immunotherapy as definitive or adjuvant therapy, neoadjuvant immunotherapy has been an area of interest due to the functional and cosmetic implications of definitive surgery. Neoadjuvant treatment may allow more function-preserving surgeries and reduce the need for postoperative radiation therapy. A hallmark phase II clinical trial provided evidence that neoadjuvant cemiplimab was effective in immunocompetent patients with stage II to IV cSCC. There was a 51% complete response rate and 68% overall response rate, with some patients achieving a reduction in disease that made surgical preservation of key functional structures possible [89]. Future studies are needed to investigate whether neoadjuvant immunotherapy is also effective in immunosuppressed patients.

## 9. Prevention and Prophylaxis

Reducing exposure to risk factors for cSCC is a key consideration in preventing cSCC in immunosuppressed patients. It is well known that UV exposure is one of the main risk factors for the development of cSCC, especially for immunosuppressed patients [90]. In solid organ transplantation patients, over 90% of skin cancer presents in areas exposed to UV light [91]. Therefore, sun protection including high-SPF, broad-spectrum sunscreen and sun-protective clothing as well as avoidance of excessive sun exposure and sunburn are critical preventative measures. These preventative resources are widely available and have low relative cost of implementation. In addition, they are known to be effective, suggesting that prevention may be the most important intervention to emphasize in immunocompromised patients.

Prophylactic retinoids have been shown to be effective in reducing the incidence of cSCC as well as treating premalignant lesions. Retinoids are analogues of vitamin A that have a chemoprophylactic effect via an unknown mechanism that probably involves immunomodulation and initiation of apoptosis [92]. Acitretin is the most common and best supported prophylactic retinoid that is used for cSCC. It is indicated in patients with a high risk of developing multiple cSCCs, such as solid organ transplant patients [92,93]. In renal transplant recipients with a history of keratotic lesions, a 6-month course of acitretin demonstrated a significant reduction in the incidence of cSCC compared with placebo in a randomized controlled trial [94]. Other studies have found similar effects in patients with other solid organ transplants and a past history of cSCCs [95]. However, life-long treatment with acitretin is necessary to maintain its effects. Discontinuation of use is also associated with a relapse in lesions [92]. 

Another drug that has been investigated for prevention of cSCC development is capecitabine, an oral prodrug of 5-fluorouracil. Studies on solid organ transplantation recipients revealed that oral capecitabine significantly reduced the incidence of cSCC and premalignant lesions in solid organ transplant patients [96,97]. With a median treatment time of over 12 years, capecitabine was well tolerated by patients. However, after discontinuation of capecitabine, some patients experienced a rebound in SCC incidence, particularly if their dose had been reduced towards the end of treatment, suggesting that continuous administration may be necessary to maintain its preventive effect [96]. Comparison of the efficacy of capecitabine with that of acitretin should be considered as a focus of future study.

In more recent years, nicotinamide has emerged as a possible chemoprophylactic agent. Nicotinamide reduces the immunosuppression caused by DNA damage from UV radiation and facilitates DNA repair. A phase III clinical trial with immunocompetent patients demonstrated that nicotinamide reduced the number of cSCC lesions by approximately 30% in comparison to the placebo [98]. However, when nicotinamide was studied in solid organ transplant recipients, it failed to produce a significant difference in cSCC counts [99].

See Table 2 for a summary of studies investigating prophylactic therapies to prevent development of cSCC and treat pre-malignant lesions.

## 10. Conclusions

Immunosuppression is a major risk factor for cSCC that is associated with more aggressive disease presentation and worse prognosis. As such, treatment of immunosuppressed patients requires special considerations. Prevention of cSCC development through reducing UV exposure, treatment of premalignant lesions, and minimization of the carcinogenicity of their immunosuppressive regimens is a key aspect of the management of these patients. Currently, the primary treatment for cSCC in immunosuppressed patients is surgery, which provides the best rates of local control. Radiation therapy and chemotherapy should only be considered as primary treatments in cases where surgery is unlikely to achieve a cure or where patients are poor surgical candidates. Either may have a role as adjuvant treatment, due to the increased likelihood of recurrence and metastasis, but future studies are necessary to better define the role of these treatment modalities for immunosuppressed patients. Immunotherapy has shown promising results in immunocompetent patients in recent years. However, evidence of its use in immunosuppressed patients is limited to case studies and small clinical trials, and there are concerns about worsening underlying conditions for these patients. Further studies to establish the safety and efficacy of immunotherapy in immunosuppressed patients are urgently needed. 

Though this review has focused on studies that recruited immunosuppressed patients for selection, it is limited by the fact that all of the treatment modalities for cSCC were primarily established in immunocompetent patients. Much of the data for certain treatment modalities in the immunosuppressed patient are still limited to small case series or retrospective reviews. In addition, there may be differences in the relative efficacy of treatments for cSCC in patients with different etiologies of immunosuppression. For instance, transplant rejection is a concern with immunotherapy in solid organ transplant recipients, but patients with HIV may have a different risk profile and response to immunotherapy treatment. Further study investigating treatment tailored to these groups is encouraged.

This review highlights the nuance and complexity involved in treating cSCC in immunosuppressed patients. There are multiple treatment modalities that should be considered, and many patients will require a combination of modalities concurrently or in sequence to optimally manage their disease. With this in mind, we recommend that when cSCC is present, providers develop an individualized treatment plan for each patient, involving prevention, prophylaxis, and one or more of the available modalities discussed in this review. When possible, patients should be evaluated by a multidisciplinary team, in order to better develop a comprehensive plan for therapy and involve the patient in decision making. Patient preferences and goals of care should be addressed in the treatment planning process.

## Figures and Tables

**Table 2:** Studies in prevention of cSCC.

Author	Study Design	Cohort	Treatment	Results/Outcomes
Bavinck et al. [28]	Randomized controlled trial	44 renal transplant recipients	Acitretin 30 mg/day × 6 months	Over 12 months, 2/19 patients in the treatment group developed new cSCC lesions and had a 13.4% decrease in keratotic lesions, while 9/19 patients in the placebo group developed new cSCC lesions and had a 28.2% increase in lesions.
Harwood et al. [29]	Retrospective study	32 organ transplant recipients	Continuous systemic retinoids 0.2 to 0.4 mg/kg/day for a minimum of 12 months	Mean reduction of 1.46 cSCC lesions developed per year after starting therapy. Statistically significant reduction in first 3 years of treatment. No serious adverse effects from therapy noted.
Jirakulaporn et al. [30]	Retrospective study	15 solid organ transplant recipients	Oral capecitabine 1 g/m^2^ BID × 14 days	13/15 patients showed reduction in incidence of new cSCC lesions with treatment, with overall incidence reuction of 0.33. One patient required dose reduction due to toxicity.
Endrizzi et al. [31]	Case series	10 solid organ transplant recipients	Oral capecitabine 0.5–1.5 g/m^2^/day × 14 days	9/10 patients showed reduction in incidence of new cSCC lesions in 12 months of treatment, with 68% mean reduction. 7/10 patients required dose adjustment due to toxicity.
Allen et al. [33]	Randomized controlled trial	158 organ transplant recipients	Nicotinamide 500 mg BID × 12 months	No significant difference noted in incidence of cSCC between groups, and no significant difference in number of adverse effects.
Hasan et al. [47]	Randomized controlled trial	40 organ transplant recipients	Topical 5% 5-fluorouracil (5-FU) vs. 5% imiquimod	After 12 months, 58% of 5-FU patients had at least 75% lasting reduction in keratotic lesions, with only 15% of sunscreen patients achieving 75% reduction.
